# Engineering small‐molecule analogues of altiratinib via CREB‐regulated transcription co‐activator 3‐target screening for the development of potent and safe topical therapeutics against skin hyperpigmentary diseases

**DOI:** 10.1002/ctm2.1625

**Published:** 2024-03-14

**Authors:** Jeong Hyeon Lee, Hongchan An, HyeJi Kwon, Su‐Jeong Lee, Young Hye Park, Ji Sun Hwang, Min Young Kim, Hayoung Hwang, Jeong Yoon Kim, Seung Jin Lee, Sung Eun Chang, Youngsup Song

**Affiliations:** ^1^ Department of Dermatology University of Ulsan College of Medicine, Asan Medical Center Seoul South Korea; ^2^ College of Pharmacy and Institute of Pharmaceutical Sciences CHA University Pocheon South Korea; ^3^ New Drug Development Center (NDDC) Daegu‐Gyeongbuk Medical Innovation Foundation (DGMIF) Daegu South Korea; ^4^ Department of Brain Science Brain Korea 21 project University of Ulsan College of Medicine, Asan Medical Center Seoul South Korea

Dear Editor,

This study aimed to develop topical therapeutics for the treatment of UV‐induced skin hyperpigmentary disorders and demonstrates how a compound identified through a robust mechanism‐based screen was successfully engineered using medicinal chemistry. CREB‐regulated transcription co‐activator 3 (CRTC3) is the critical upstream regulator of MITF, which is a central regulator of melanogenesis by modulating tyrosinase, tyrosinase‐related protein‐1 (TYRP1), and dopamine tautomerase (DCT) in ultraviolet (UV) or cyclic adenosine monophosphate that is forskolin (FSK)‐induced pathways.^1^, ^2^, ^3^ Building upon the discovery of a tunable and reversible regulation of MITF by CRTC3 nuclear shuttle,^4^ we established a screening tool for CRTC3 inhibitors and performed a high‐throughput screening of small molecules targeting CRTC3. While altiratinib (ALT) was screened and demonstrated a dose‐dependent inhibitory action on CRTC3 activity and melanogenesis up to 1 µM, cytotoxicity emerged at higher concentrations (Figure S1A). There is a need to enhance both its safety margin and efficacy to address the limitations of anti‐pigmentation topical drugs or cosmeceuticals.^5^ Since, ALT was developed to target multiple‐growth factor receptors, c‐Met, VEGFR2 and TrKA,^6^ we explored the melanin‐inhibiting properties of other functionally comparable inhibitors, including sorafenib, foretinib and cabozantinib. While sorafenib was the only compound that exhibited a dose‐dependent reduction in melanin content in mouse melanocytes (Mel‐Ab), its efficacy was inferior to that of ALT. Furthermore, all showed cytotoxicity in Mel‐Ab and did not have anti‐melanogenesis effects on normal human melanocytes (NHM) with more pronounced cytotoxicity (Figure S1B–E).

To heighten potency, safety and skin barrier permeability for human skin application,^7^ we designed new compounds using a bioisosteric replacement strategy that incorporated different ring systems of ALT.^8^ We selected representative analogues from each bicyclic group (6, indazole; 7, benzoxazole; 8, benzothiazole) while considering their cytotoxicities and potencies, resulting in the creation of a series of ALT analogues (Figure S2A–D). Subsequently, we screened ALT analogues that exhibited no more than 10% cytotoxicity at concentrations ten times higher than their effective concentration and a concentration‐dependent melanin‐inhibiting effect in both Mel‐Ab and B16F10 mouse melanoma cells. Through this process, we identified ALT6a, ALT7a, and ALT8c which demonstrated comparable to or better than ALT in inhibition of FSK‐stimulated melanogenesis without cytotoxicity (Figures S3A,B and S4A,B). Generally, NHM exhibited higher sensitivity than Mel‐Ab to cytotoxicity at a concentration of 10 µM, (Figure 1A). ALT6a and ALT7a consistently demonstrated anti‐melanogenesis activity, while ALT8c, which reduced melanin content in Mel‐Ab and B16F10, did not inhibit melanogenesis in NHM (Figure 1B) positioning ALT7a as top and ALT6a as second top candidates that exert a dose‐dependent reduction in melanin content with no or minimal cytotoxicity, respectively, in all three cells (Figure 1A,B and Figures S3 and S4).

**FIGURE 1:**
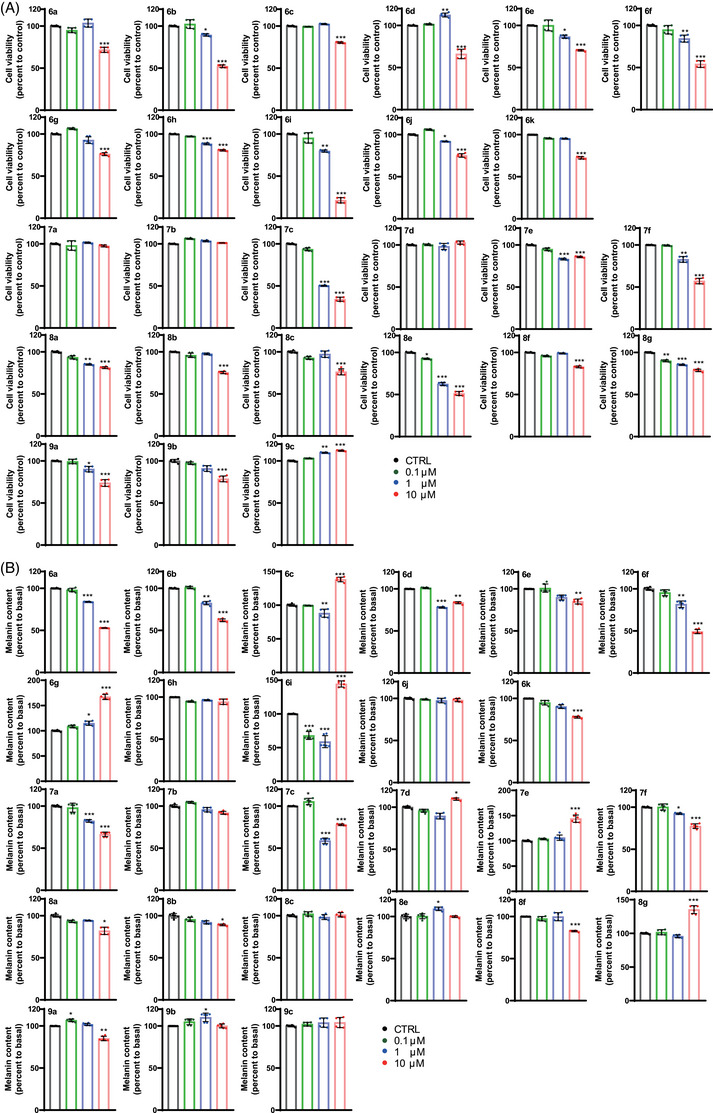
**Effect of altiratinib analogues on cell viability and melanogenesis of normal human melanocytes**. (A) Cellular viability of normal human melanocytes (NHM) after 72 h of treatment with 0.1–10 µM of ALT analogue series 6, 7, 8 and 9 assessed by the MTT assay. (B) The melanin content of NHM treated with 0.1−10 µM of ALT analogue series 6, 7, 8 and 9 for 72 h measured and displayed as a percent change compared with the vehicle‐treated controls.

ALT6a and ALT7a demonstrated superior efficacy and a wider safety margin to ALT, although the safety margin of ALT6a was slightly narrower than that of ALT7a in NHM (Figure 2A). In NHM, ALT6a and ALT7a displayed a more efficient reduction in melanin content than ALT (Figure 2B), whereas the efficacy of melanin reduction achieved by ALT6a and ALT7a was comparable to that observed with ALT in Mel‐Ab and primary melanocytes cultured from KRT14‐stem cell factor (SCF)‐epidermal humanized mice^9^ (Figure 2C and Figure S7A–C). ALT, ALT6a and ALT7a for 72 h resulted in a comparable decrease in the FSK‐induced elevation of cellular tyrosinase activity in Mel‐Ab (Figure 2D), whereas in tube mushroom tyrosinase activity (Figure 2E) and 2 h‐short‐term cellular tyrosinase activity remained unaffected (Figure S6).

**FIGURE 2:**
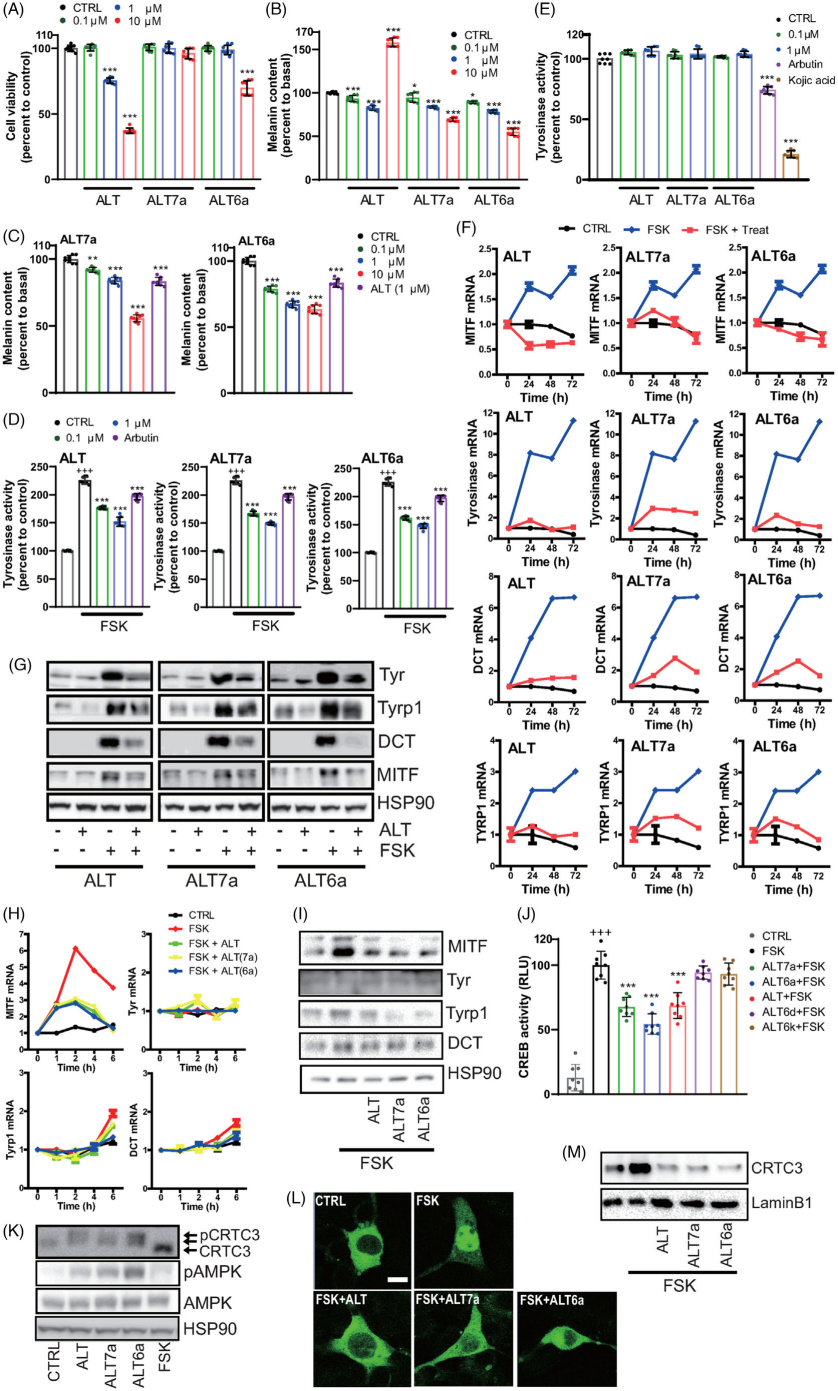
Improvement of safety profiles and suppression of melanogenesis of two novel altiratinib analogues through the phosphorylation‐dependent inhibition of CRTC3 nuclear translocation‐mediated downregulation of the expression of genes involved in melanin biosynthesis. (A) Cell viability and (B) melanin content of primary human melanocytes treated with 0.1, 1, and 10 µM of novel ALT analogues (ALT7a, ALT6a) or ALT for 72 h. (C) Melanin content of K14‐SCF mouse‐derived primary melanocytes treated with 0.1, 1 and 10 µM of ALT analogues (ALT7a, ALT6a) and ALT for 72 h. (D) Tyrosinase activity of Mel‐Ab cells after treatment with the vehicle (CTRL), altiratinib (ALT), or ALT analogues (ALT7a, ALT6a) plus FSK for 72 h. (E) In vitro mushroom tyrosinase activity with ALT, ALT7a and ALT6a. Arbutin and kojic acid were used as positive controls as direct inhibitor of tyrosinase (F) Expression levels of melanogenic genes at the mRNA level (24–72 h) and (G) protein level (72 h) in Mel‐Ab cells treated with the vehicle (CTRL), FSK, or ALT analogues (ALT7a, ALT6a, or ALT) plus FSK as revealed by qRT‐PCR and Western blotting, respectively. (H) Short‐term effects of FSK and altiratinib (ALT) or ALT analogues (ALT7a, ALT6a) + FSK treatment (1−6 h) on the expression levels of melanogenic gene mRNAs. (I) Short‐term effects of FSK and altiratinib (ALT) or ALT analogues (ALT7a, ALT6a) + FSK treatment (8 h) on protein levels of melanogenic genes. (J) Effect of altiratinib (ALT) or ALT analogues (ALT7a, ALT6a) on the transcriptional activity of CRTC3/CREB using CREB target‐based promoter reporter assays. (K) Phosphorylation status of CRTC3 and AMPK by immunoblotting in Mel‐Ab cells following 1 h of altiratinib (ALT) or ALT analogue (ALT7a, ALT6a) treatment. (L) Representative microscopic images of the subcellular localization of CRTC3 1 h after treatment with altiratinib (ALT), ALT analogues (ALT7a, ALT6a), FSK in Mel‐Ab cells expressing CRTC3‐EGFP. Bar = 10 µm. (M) Representative Western blot images of nuclear CRTC3 protein levels after 1 h of treatment with altiratinib (ALT), ALT analogues (ALT7a, ALT6a), and FSK in Mel‐Ab cells.

Then, we investigated whether the decreased melanin content and cellular tyrosinase activity by ALT6a and ALT7a were linked to a reduction of pigmentation genes. 72 h of FSK‐stimulated upregulation of mRNA and protein levels of melanogenesis genes were significantly attenuated by pretreatment with ALT or ALT6a and ALT7a (Figure 2F,G), whereas short‐term treatment (up to 8 hours) with these compounds selectively inhibited mRNA and protein expression levels of MITF, but not those of tyrosinase, TYRP1, and DCT (Figure 2H,I). Mechanistically, they suppressed the FSK‐stimulated transcriptional activity of CREB (Figure 2J), which was attributed to the phosphorylation‐dependent intracellular localization of CRTC3 by ALT6a and ALT7a (Figure 2K–M and Figure S7). Exploring relevant kinase profiles suggests that AMPK and ERK are presumed to be responsible for CRTC3 hyperphosphorylation mediated by ALT, ALT7a and ALT6a (Figure 2K and Figure S8). Interestingly, while ALT downregulated JNK and AKT activity, ALT7a and ALT6a did not affect them, potentially contributing to the differential effects on efficacy and cell viability between ALT and ALT7a or ALT6a (Figure S8). To evaluate the potential of ALT6a and ALT7a as skin topicals, a skin parallel artificial membrane permeability assay (PAMPA) was performed.^10^ In comparison to ALT, ALT6a and ALT7a exhibited enhanced skin permeability (Figure 3A). We further assessed the efficacy and skin tissue toxicity of ALT 6a and ALT7a in KRT14‐SCF mice and UVB‐irradiated human skin explants. The topical application of ALT6a and ALT7a to the tails of KRT14‐SCF mice more potently reduced epidermal melanin (Figure 3B–D) and tyrosinase protein levels (Figure 3E) than ALT. In UVB‐irradiated human skin culture for 24 hours, UVB triggered the nuclear translocation of CRTC3 in epidermal melanocytes, which was attenuated by topical exposure to ALT, ALT6a and ALT7a (Figure 3F). UVB‐stimulated melanin deposition (Figure 3G,H) and upregulation of melanogenesis gene expression were more potently reduced by ALT6a and ALT7a (Figure 3I) than by ALT. This suggests that the enhanced anti‐pigmentation potency observed in mouse skin and human skin for ALT6a and ALT7a, especially at lower doses, may likely be attributed to their improved skin barrier permeability due to modifications in the chemical structure. These findings highlight the potential of refining topical drugs with enhanced skin permeability alongside efficacy and safety through medicinal chemistry.

**FIGURE 3:**
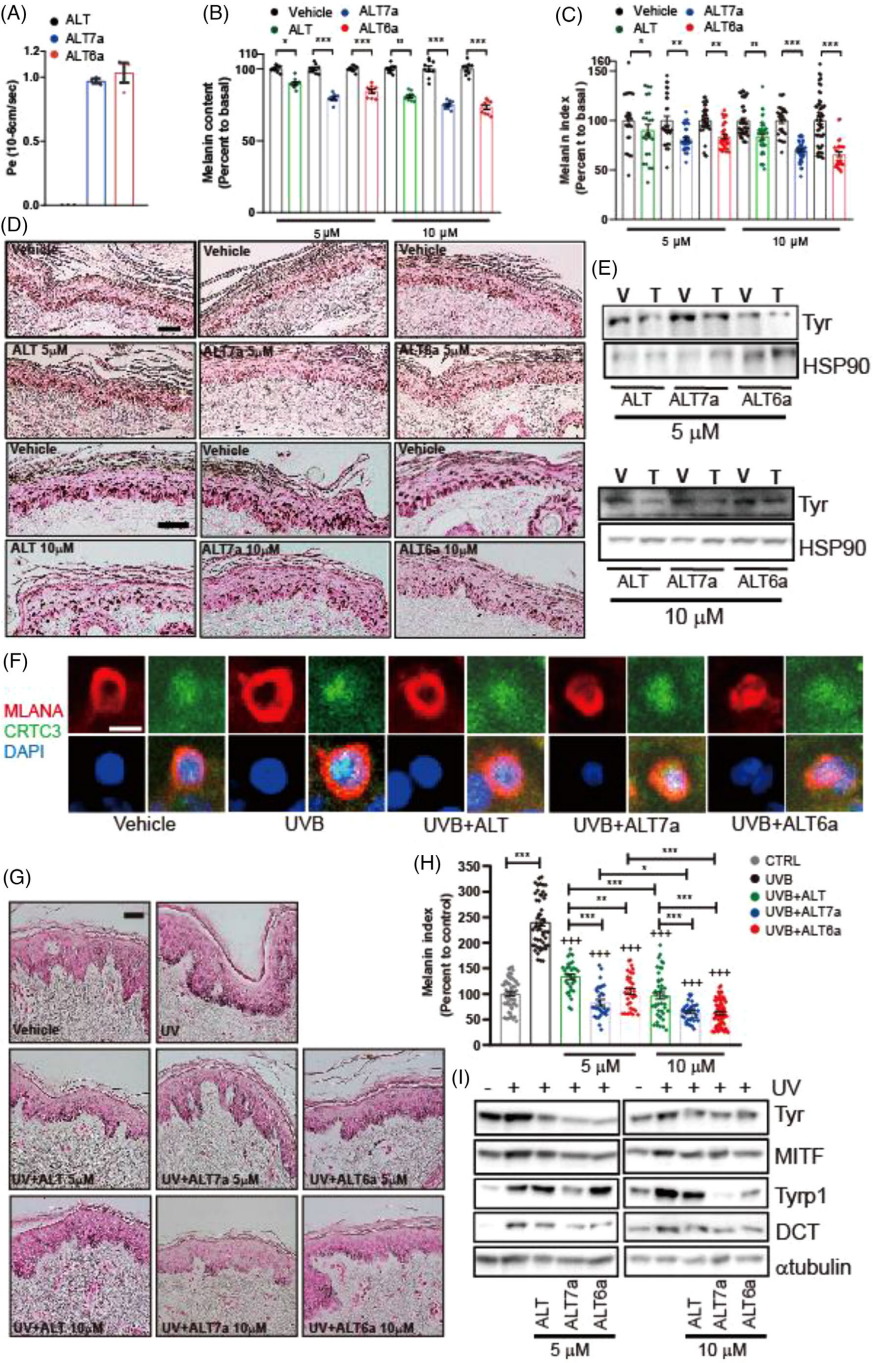
**Suppression of melanogenesis in in vivo mouse and ex vivo human skin culture by altiratinib analogues**. (A) Comparison of the predicted skin permeability of altiratinib (ALT) and ALT analogues (ALT7a, ALT6a) assessed by the skin PAMPA experiment. (B) Melanin content, (C) melanin index, and (D) representative images of Fontana–Masson‐stained paraffin‐embedded tail skin sections of KRT14‐SCF transgenic mice treated with the vehicle (CTRL), 10 µM altiratinib (ALT), or 10 µM ALT analogues (ALT7a, ALT6a). Bars = 50 µm. (E) Tyrosinase protein level in mouse tail tissue exposed to the vehicle (CTRL), 5 or 10 µM altiratinib (ALT), or ALT analogues (ALT7a, ALT6a) as assessed by Western blotting. (F) Immunofluorescence staining of MLANA (red), CRTC3 (green) antibody with nuclear DAPI (blue) staining of human skin 24 h after UVB or UVB + ALT, or ALT analogue (ALT7a, ALT6a) treatment. Bar = 5 µm (G) Representative images and (H) melanin index of Fontana–Masson‐stained paraffin‐embedded sections treated with the vehicle (CTRL), UVB + vehicle (UV), or UVB + 5 or 10 µM altiratinib (ALT) or ALT analogues (ALT7a, ALT6a). Bars = 50 µm. (I) Protein expression level of melanogenic genes in the human skin tissue exposed to the vehicle alone (CTRL), UVR + vehicle (UV), or UVB + 5 or 10 µM altiratinib (ALT) or ALT analogues (ALT7a, ALT6a) as assessed by Western blotting.

## CONCLUSION

Numerous skin‐lightening topicals targeting tyrosinase have been developed but none were safe and efficacious enough.^2^, ^3^, ^5^ As our previous studies demonstrated,^1^ targeting CRTC3 can attain the key treatment goal of hyperpigmentation disorders, not the death of melanocytes but the homeostatic modulation of melanin synthesis.^5^ We showed that ALT6a and ALT7a serve as excellent examples for drug repositioning and small‐molecule fabrication, highlighting their potential for developing highly potent and non‐toxic topicals for the treatment of extremely common UV and photo‐ageing‐associated hyperpigmentary diseases.

## AUTHOR CONTRIBUTIONS

Conceptualization and design of the experiments, Hongchan An, Sung Eun Chang and Youngsup Song; methodology, Jeong Hyeon Lee, Hongchan An, Hye Ji Kwon, Seung Jin Lee, Hayoung Hwang, Sung Eun Chang and Youngsup Song; investigation and visualization and curation of the data, Jeong Hyeon Lee, Hye Ji Kwon, Seung Jin Lee, Young Hye Park, Ji sun Hwang, Min Young Kim, Hayoung Hwang, JeongYoon Kim and Seung Jin Lee; supervision of the experiment, Hongchan An, Sung Eun Chang and Youngsup Song; Writing the original draft, Jeong Hyeon Lee, Hongchan An, Sung Eun Chang and Youngsup Song; Edition of the manuscript, Sung Eun Chang and Youngsup Song.

## CONFLICT OF INTEREST STATEMENT

The authors declare no conflict of interest.

## FUNDING INFORMATION

This study was supported by a grant from the National Research Foundation of Korea (2020R1A4A4079708, RS‐2023‐00208426, RS‐2023‐00246165) and Ministry of Health & Welfare, Republic of Korea (HP23C0072).

## ETHICS STATEMENT

The utilization of human skin tissue for ex vivo research was granted approval by the Institutional Review Board (IRB) of Asan Medical Center (IRB no. 2020‐0091). This human skin tissue was obtained from individuals who had been informed, provided voluntary consent, and had undergone neck or abdomen reduction surgery at Asan Medical Center under the same IRB number (2020‐0091). All animal studies were conducted according to the protocol approved by the Institutional Animal Care and Use Committee (IACUC no 2020‐02‐248) of the Asan Medical Center, Seoul, Korea.

## Supporting information

Supporting Information

## Data Availability

The datasets generated during and/or analyzed during the current study are available from the corresponding author upon reasonable request.
