# Supplementary material for: Engineering small‐molecule analogues of altiratinib via CREB‐regulated transcription co‐activator 3‐target screening for the development of potent and safe topical therapeutics against skin hyperpigmentary diseases
Source: Clin Transl Med. 2024 Mar 14;14(3):e1625. doi: 10.1002/ctm2.1625 (PMC10938064; doi:10.1002/ctm2.1625)
Supplement: Supplementary file 1 — Supporting Information [file CTM2-14-e1625-s001.docx]

**Supplementary Methods**

**Synthesis of ALT analogs**

**General Procedures:** All nuclear magnetic resonance (NMR) experiments were conducted using an Avance III 400 MHz NMR spectrometer equipped with a 5-mm broadband-observed probe head (Bruker, Billerica, MA, USA). The NMR spectrum was optimized using the Bruker Topspin 3.1 software, and all parameters were set in it. Mass spectra were measured in the positive electrospray ionization mode on the LCMS system (Shimadzu, Tokyo, Japan, or Thermo Fisher Scientific, MA, USA). Column chromatography was performed using a CombiFlash® Rf system with RediSep® Rf (Teledyne Isco, Lincoln, NE, USA). The final compounds were further purified by preparative high-performance liquid chromatography (HPLC) on Kinetex® 5 μm Biphenyl 100 Å (column tube, 250 mm × 21.2 mm ID; GX-281 HPLC System, Gilson, Middleton, WI, USA) with ACN/H_2_O as eluent. Reverse-phase HPLC was conducted on an Agilent Technologies 1290 Infinity, which was equipped with a C18 column (Kinetex® 1.7 µm EVO C18, 100 × 2.1 mm). Mobile phases A and B were water with 0.1% TFA and acetonitrile with 0.1% TFA, respectively. A gradient of 20%−100% B was run at a flow rate of 0.6 mL/min over 5 min. The purity of the target compounds was determined to be >95% by analytical HPLC. Starting materials were obtained from commercial suppliers including Aldrich (St. Louis, MO, USA) and Alfa Aesar (Ward Hill, MA, USA). Solvents were obtained from Aldrich and were used without further purification unless noted otherwise.

**Chemistry**

**2-chloro-4-(2,5-difluoro-4-nitrophenoxy)pyridine (3)**

A suspension of sodium hydride (60% in dispersion, 40.1 mg, 1.00 mmol) in DMF (430.0 μL) was added with a solution of 2-chloropyridin-4-ol (100.0 mg, 0.77 mmol) (1000.0 μL) at 0 ℃. The mixture was stirred for 30 min, and a solution of 1,2,4-trifluoro-5-nitrobenzene (150,0 mg, 0.85 mmol) in DMF (500.0 μL) was added at the same temperature. The resulting mixture was warmed to room temperature and stirred overnight. To remove the majority of DMF in the mixture, the reaction mixture was concentrated under reduced pressure and was then partitioned between ethyl acetate and 10% aqueous lithium chloride. The precipitate formed was removed via suction filtration, and the layers were then separated. The organic layer was washed with additional 10% aqueous lithium chloride (×2), saturated aqueous sodium bicarbonate, and brine. The organic layer was dried over anhydrous MgSO_4_ and concentrated under reduced pressure. The residue was purified by silica gel chromatography (25% EtOAc/*n*-hexane) to afford 125.8 mg (57%) of 2-chloro-4-(2,5-difluoro-4-nitrophenoxy)pyridine as a yellow solid: ^1^H NMR (400 MHz, CDCl_3_) *δ* 8.39 (d, *J* = 5.7 Hz, 1H), 8.06 (dd, *J* = 9.3, 6.8 Hz, 1H), 7.14 (dd, *J* = 10.3, 6.4 Hz, 1H), 6.96 (d, *J* = 2.2 Hz, 1H), 6.89 (dd, *J* = 5.7, 2.2 Hz, 1H); LCMS (ESI), m/z = 328.07 [M+1+CH_3_CN]^+^.

***N*-(4-(2,5-difluoro-4-nitrophenoxy)pyridin-2-yl)cyclopropanecarboxamide (4)**

A mixture of Pd_2_(dba)_3_ (61.3 mg, 0.07 mmol), rac-BINAP (83.0 mg, 0.13 mmol), and K_3_PO_4_ (592.0 mg, 2.79 mmol) was added with 2-chloro-4-(2,5-difluoro-4-nitrophenoxy)pyridine (320.0 mg, 1.12 mmol) in 1,4-dioxane (3720.0 μL) and cyclopropanecarboxamide (380.0 mg, 4.47 mmol) in 1,4-dioxane (3720.0 μL). The reaction mixture was heated to 100 ℃ and stirred overnight. The reaction mixture was cooled to room temperature and filtered through a Celite pad. The filtrate was concentrated under reduced pressure, and the residue was purified by silica gel chromatography (25%–30% EtOAc/*n*-hexane) to afford 150.1 mg (37%) of *N*-(4-(2,5-difluoro-4-nitrophenoxy)pyridin-2-yl)cyclopropanecarboxamide as a yellow solid: ^1^H NMR (400 MHz, CDCl_3_) *δ* 8.26-8.22 (m, 2H), 8.02 (dd, *J* = 9.5, 6.8 Hz, 1H), 7.90 (d, *J* = 2.3 Hz, 1H), 7.05 (dd, *J* = 10.7, 6.5 Hz, 1H), 6.73 (dd, *J* = 5.7, 2.4 Hz, 1H), 1.57–1.51 (m, 1H), 1.10–1.06 (m, 2H), 0.94-0.89 (m, 2H); LCMS (ESI), m/z = 336.08 [M+1]^+^.

***N*-(4-(4-amino-2,5-difluorophenoxy)pyridin-2-yl)cyclopropanecarboxamide (5)**

A solution of *N*-(4-(2,5-difluoro-4-nitrophenoxy)pyridin-2-yl)cyclopropanecarboxamide (150.0 mg, 0.45 mmol) in methanol (2237.0 μL) and THF (2237.0 μL) was added with NH_4_Cl (239.0 mg, 4.47 mmol) and zinc dust (293.0 mg, 4.47 mmol) at 0 ℃, and the mixture was stirred for 30 min at the same temperature. The resulting mixture was warmed to room temperature and stirred overnight. The reaction mixture was filtered through a Celite pad, which was washed well with methanol. The filtrate was concentrated under reduced pressure, and the residue was partitioned between ethyl acetate and water. The organic layer was washed with additional water and brine, dried over anhydrous MgSO_4_, and concentrated under reduced pressure. The residue was purified by NH_2_ silica gel chromatography (30% EtOAc/*n*-hexane) to afford 113.0 mg (83%) of *N*-(4-(4-amino-2,5-difluorophenoxy)pyridin-2-yl)cyclopropanecarboxamide as a white solid: ^1^H NMR (400 MHz, DMSO-*d*_6_) *δ* 10.83 (s, 1H), 8.17 (d, *J* = 5.7 Hz, 1H), 7.61 (d, *J* = 2.3 Hz, 1H), 7.17 (dd, *J* = 11.2, 7.5 Hz, 1H), 6.75–6.67 (m, 2H), 5.51 (s, 2H), 2.00-1.94 (m, 1H), 0.79–0.77 (m, 4H); LCMS (ESI), m/z = 306.11 [M+1]^+^.

**General procedure A for amide coupling reaction**

A solution of *N*-(4-(4-amino-2,5-difluorophenoxy)pyridin-2-yl)cyclopropanecarboxamide (1.0 equiv) in DMF was added with acid (1.2 equiv), *N*,*N*-diisopropylethylamine (5.0 equiv) and TBTU or HATU (1.2 equiv) as a coupling reagent (TBTU; *O*-(benzotriazol-1-yl)-*N*,*N*,*N*',*N*'-tetramethyluronium tetrafluoroborate or HATU; 1-[Bis(dimethylamino)methylene]-1*H*-1,2,3-triazolo[4,5-*b*]pyridinium 3-oxid hexafluorophosphate). The reaction mixture was stirred at room temperature. The reaction mixture was diluted with ethyl acetate and washed with 10% aqueous lithium chloride (×3) and brine. The organic layer was dried over anhydrous MgSO_4_ and concentrated under reduced pressure.

**General procedure B for amide coupling reaction**

A solution of *N*-(4-(4-amino-2,5-difluorophenoxy)pyridin-2-yl)cyclopropanecarboxamide (1.0 equiv) in THF was added with acid (1.2 equiv), 1-hydroxybenzotriazole (1.1 equiv), and DCC (1.1 equiv) as a coupling reagent (DCC; *N*,*N*'-dicyclohexylcarbodiimide). The reaction mixture was stirred at 60 ℃. The reaction mixture was diluted with ethyl acetate and washed with 10% aqueous lithium chloride (×3) and brine. The organic layer was dried over anhydrous MgSO_4_ and concentrated under reduced pressure.

***N*-(4-(4-(2-(1*H*-indazol-3-yl)acetamido)-2,5-difluorophenoxy)pyridin-2-yl)cyclopropane yl)cyclopropanecarboxamide (6a)**

The amide coupling reaction of 2-(1*H*-indazol-3-yl)acetic acid (17.3 mg, 0.10 mmol) using HATU for 42 h via general procedure A was followed by NH_2_ silica gel chromatography (60% EtOAc/*n*-hexane) to afford 1.4 mg (5%) of *N*-(4-(4-(2-(1*H*-indazol-3-yl)acetamido)-2,5-difluorophenoxy)pyridin-2-yl)cyclopropanecarboxamide as a white solid (99% purity): ^1^H NMR (400 MHz, CD_3_OD) *δ* 8.19–8.12 (m, 2H), 7.83 (d, *J* = 8.2 Hz, 1H), 7.70 (d, *J* = 2.3 Hz, 1H), 7.53 (d, *J* = 8.5 Hz, 1H), 7.44–7.40 (m, 1H), 7.25–7.16 (m, 2H), 6.70 (dd, *J* = 5.8, 2.4 Hz, 1H), 4.21 (s, 2H), 1.89–1.83 (m, 1H), 0.96–0.85 (m, 4H); LCMS (ESI), m/z = 464.23 [M+1]^+^.

***N*-(4-(2,5-difluoro-4-(2-(5-fluoro-1*H*-indazol-3-yl)acetamido)phenoxy) pyridin-2-yl)cyclopropanecarboxamide (6b)**

The amide coupling reaction of 2-(5-fluoro-1*H*-indazol-3-yl)acetic acid (2-(benzo[*d*]oxazol-2-yl)acetic acid (19.0 mg, 0.10 mmol) using TBTU for 76 h via general procedure A was followed by prep-HPLC (20%–100% water/acetonitrile with 0.1% TFA) to afford 2.4 mg (6%) of *N*-(4-(2,5-difluoro-4-(2-(5-fluoro-1*H*-indazol-3-yl)acetamido)phenoxy)pyridin-2-yl)cyclopropanecarboxamide as a colorless oil (96% purity): ^1^H NMR (400 MHz, CD_3_OD) *δ* 8.26–8.21 (m, 2H), 7.55–7.48 (m, 2H), 7.38 (dd, *J* = 10.5, 7.1 Hz, 1H), 7.24 (td, *J* = 9.1, 2.4 Hz, 1H), 7.08–7.06 (m, 2H), 4.19 (s, 2H), 1.86–1.80 (m, 1H), 1.11–1.00 (m, 4H); LCMS (ESI), m/z = 482.08 [M+1]^+^.

***N*-(4-(2,5-difluoro-4-(2-(5-methoxy-1*H*-indazol-3-yl)acetamido)phenoxy)pyridin-2-yl)cyclopropanecarboxamide (6c)**

The amide coupling reaction of 2-(5-methoxy-1*H*-indazol-3-yl)acetic acid (32.4 mg, 0.16 mmol) using HATU for 21 h via general procedure A was followed by silica gel chromatography (75% EtOAc/*n*-hexane) to afford 12.8 mg (19%) of *N*-(4-(2,5-difluoro-4-(2-(5-methoxy-1*H*-indazol-3-yl)acetamido)phenoxy)pyridin-2-yl)cyclopropanecarboxamide as a dark orange solid (97% purity): ^1^H NMR (400 MHz, DMSO-*d*_6_) *δ* 12.73 (s, 1H), 10.90 (s, 1H), 10.36 (s, 1H), 8.21 (d, *J* = 5.7 Hz, 1H), 8.11 (dd, *J* = 12.4, 7.3 Hz, 1H), 7.66 (d, *J* = 2.3 Hz, 1H), 7.55 (dd, *J* = 10.9, 7.4 Hz, 1H), 7.40 (d, *J* = 9.0 Hz, 1H), 7.25 (d, *J* = 2.0 Hz, 1H), 7.01 (dd, *J* = 9.0, 2.3 Hz, 1H), 6.75 (dd, *J* = 5.7, 2.4 Hz, 1H), 4.12 (s, 2H), 3.78 (s, 3H), 2.00–1.94 (m, 1H), 0.81–0.72 (m, 4H); LCMS (ESI), m/z = 493.90 [M+1]^+^.

***N*-(4-(4-(2-(benzo[*d*]isoxazol-3-yl)acetamido)-2,5-difluorophenoxy) pyridin-2-yl)cyclopropanecarboxamide (6d)**

The amide coupling reaction of 2-(benzo[*d*]isoxazol-3-yl)acetic acid (17.4 mg, 0.10 mmol) using TBTU for 22 h via general procedure A was followed by NH_2_ silica gel chromatography (30% EtOAc/*n*-hexane) to afford 12.6 mg (32%) of *N*-(4-(4-(2-(benzo[*d*]isoxazol-3-yl)acetamido)-2,5-difluorophenoxy)pyridin-2-yl)cyclopropanecarboxamide as a pale yellow solid (95% purity): ^1^H NMR (400 MHz, CDCl_3_) *δ* 8.50 (s, 1H), 8.36–8.30 (m, 2H), 8.12 (d, *J* = 5.8 Hz, 1H), 7.81 (d, *J* = 8.0 Hz, 1H), 7.75 (d, *J* = 2.2 Hz, 1H), 7.65–7.60 (m, 2H), 7.42–7.38 (m, 1H), 6.96 (dd, *J* = 10.5, 6.9 Hz, 1H), 6.59 (dd, *J* = 5.8, 2.3 Hz, 1H), 4.20 (s, 2H), 1.54–1.48 (m, 1H), 1.07–1.03 (m, 2H), 0.91–0.84 (m, 2H); LCMS (ESI), m/z = 465.22 [M+1]^+^.

***N*-(4-(4-(2-(benzo[*d*]isothiazol-3-yl)acetamido)-2,5-difluorophenoxy) pyridin-2-yl)cyclopropanecarboxamide (6e)**

The amide coupling reaction of 2-(benzo[*d*]isothiazol-3-yl)acetic acid (30.4 mg, 0.16 mmol) using HATU for 48 h via general procedure A was followed by NH_2_ silica gel chromatography (40% EtOAc/*n*-hexane) to afford 16 mg (25%) of *N*-(4-(4-(2-(benzo[*d*]isothiazol-3-yl)acetamido)-2,5-difluorophenoxy)pyridin-2-yl)cyclopropanecarboxamide as a pale yellow solid (97% purity): ^1^H NMR (400 MHz, DMSO-*d*_6_) *δ* 10.90 (s, 1H), 10.51 (s, 1H), 8.23–8.19 (m, 3H), 8.11 (dd, *J* = 12.4, 7.2 Hz, 1H), 7.67–7.51 (m, 4H), 6.75 (dd, *J* = 5.7, 2.4 Hz, 1H), 4.41 (s, 2H), 2.01–1.93 (m, 1H), 0.81–0.74 (m, 4H); LCMS (ESI), m/z = 481.12 [M+1]^+^.

***N*-(4-(4-(2-(1*H*-indol-3-yl)acetamido)-2,5-difluorophenoxy)pyridin-2-yl)cyclopropanecarboxamide (6f)**

The amide coupling reaction of 2-(1*H*-indol-3-yl)acetic acid (27.5 mg, 0.16 mmol) using HATU for 17 h via general procedure A was followed by NH_2_ silica gel chromatography (60% EtOAc/*n*-hexane) to afford 4 mg (6%) of *N*-(4-(4-(2-(1*H*-indol-3-yl)acetamido)-2,5-difluorophenoxy)pyridin-2-yl)cyclopropanecarboxamide as a pale yellow gum (96% purity): ^1^H NMR (400 MHz, CDCl_3_) *δ* 8.39–8.31 (m, 3H), 8.09 (d, *J* = 5.8 Hz, 1H), 7.73–7.67 (m, 2H), 7.63 (d, *J* = 7.7 Hz, 1H), 7.44 (d, *J* = 8.1 Hz, 1H), 7.31–7.26 (m, 2H), 7.23–7.18 (m, 1H), 6.82 (dd, *J* = 10.6, 6.9 Hz, 1H), 6.56 (dd, *J* = 5.8, 2.4 Hz, 1H), 3.95 (s, 2H), 1.54–1.47 (m, 1H), 1.07–1.02 (m, 2H), 0.89–0.83 (m, 2H); LCMS (ESI), m/z = 463.14 [M+1]^+^.

***N*-(4-(4-(2-(benzofuran-3-yl)acetamido)-2,5-difluorophenoxy)pyridin-2-yl)cyclopropanecarboxamide (6g)**

The amide coupling reaction of 2-(benzofuran-3-yl)acetic acid (27.7 mg, 0.16 mmol) using HATU for 30 h via general procedure A was followed by NH_2_ silica gel chromatography (45% EtOAc/*n*-hexane) to afford 8.8 mg (14%) of *N*-(4-(4-(2-(benzofuran-3-yl)acetamido)-2,5-difluorophenoxy)pyridin-2-yl)cyclopropanecarboxamide as a white solid (98% purity): ^1^H NMR (400 MHz, DMSO-*d*_6_) *δ* 10.90 (s, 1H), 10.34 (s, 1H), 8.21 (d, *J* = 5.7 Hz, 1H), 8.10 (dd, *J* = 12.3, 7.2 Hz, 1H), 7.93 (s, 1H), 7.69 (d, *J* = 7.0 Hz, 1H), 7.66 (d, *J* = 2.2 Hz, 1H), 7.59–7.52 (m, 2H), 7.36–7.25 (m, 2H), 6.75 (dd, *J* = 5.7, 2.4 Hz, 1H), 3.91 (s, 2H), 1.99–1.96 (m, 1H), 0.78 (d, *J* = 5.5 Hz, 4H); LCMS (ESI), m/z = 464.00 [M+1]^+^.

***N*-(4-(4-(2-(benzo[*b*]thiophen-3-yl)acetamido)-2,5-difluorophenoxy)pyridin-2-yl)cyclopropanecarboxamide (6h)**

The amide coupling reaction of 2-(benzo[*b*]thiophen-3-yl)acetic acid (30.2 mg, 0.16 mmol) using HATU for 31 h via general procedure A was followed by NH_2_ silica gel chromatography (25% EtOAc/*n*-hexane) to afford 14.3 mg (22%) of *N*-(4-(4-(2-(benzo[*b*]thiophen-3-yl)acetamido)-2,5-difluorophenoxy)pyridin-2-yl)cyclopropanecarboxamide as a white solid (97% purity): ^1^H NMR (400 MHz, DMSO-*d*_6_) *δ* 10.93 (s, 1H), 10.40 (s, 1H), 8.21 (d, *J* = 5.7 Hz, 1H), 8.12 (dd, *J* = 12.4, 7.2 Hz, 1H), 8.00 (d, *J* = 7.5 Hz, 1H), 7.91 (d, *J* = 7.6 Hz, 1H), 7.66 (d, *J* = 2.3 Hz, 1H), 7.64 (s, 1H), 7.57 (dd, *J* = 10.9, 7.4 Hz, 1H), 7.46–7.37 (m, 2H), 6.75 (dd, *J* = 5.7, 2.4 Hz, 1H), 4.08 (s, 2H), 2.00–1.94 (m, 1H), 0.78 (d, *J* = 6.1 Hz, 4H); LCMS (ESI), m/z = 480.00 [M+1]^+^.

***N*-(4-(4-(3-(1*H*-indol-3-yl)propanamido)-2,5-difluorophenoxy)pyridin-2-yl)cyclopropanecarboxamide (6i)**

The amide coupling reaction of 3-(1*H*-indol-3-yl)propanoic acid (29.8 mg, 0.16 mmol) using HATU for 18 h via general procedure A was followed by NH_2_ silica gel chromatography (50% EtOAc/*n*-hexane) to afford 27.1 mg (42%) of *N*-(4-(4-(3-(1*H*-indol-3-yl)propanamido)-2,5-difluorophenoxy)pyridin-2-yl)cyclopropanecarboxamide as a pale yellow solid (96% purity): ^1^H NMR (400 MHz, DMSO-*d*_6_) *δ* 10.92 (s, 1H), 10.79 (s, 1H), 10.03 (s, 1H), 8.21 (d, *J* = 5.7 Hz, 1H), 8.13 (dd, *J* = 12.4, 7.2 Hz, 1H), 7.65 (d, *J* = 2.4 Hz, 1H), 7.59–7.49 (m, 2H), 7.33 (d, *J* = 8.0 Hz, 1H), 7.15 (d, *J* = 2.2 Hz, 1H), 7.09–7.04 (m, 1H), 7.01–6.96 (m, 1H), 6.75 (dd, *J* = 5.7, 2.4 Hz, 1H), 3.04–3.00 (m, 2H), 2.83–2.79 (m, 2H), 2.00–1.94 (m, 1H), 0.80–0.75 (m, 4H); LCMS (ESI), m/z = 477.16 [M+1]^+^.

***N*-(4-(2,5-difluoro-4-(2-(3-oxoisoindolin-1-yl)acetamido)phenoxy)pyridin-2-yl)cyclopropanecarboxamide (6j)**

The amide coupling reaction of 2-(3-oxoisoindolin-1-yl)acetic acid (22.6 mg, 0.12 mmol) using DCC for 72 h via general procedure B was followed by NH_2_ silica gel chromatography (3% MeOH/CH_2_Cl_2_) to afford 8 mg (17%) of *N*-(4-(2,5-difluoro-4-(2-(3-oxoisoindolin-1-yl)acetamido)phenoxy)pyridin-2-yl)cyclopropanecarboxamide as a pale yellow solid (98% purity): ^1^H NMR (400 MHz, DMSO-*d*_6_) *δ* 10.94 (s, 1H), 10.16 (s, 1H), 8.75 (s, 1H), 8.26–8.16 (m, 2H), 7.70–7.59 (m, 4H), 7.59–7.48 (m, 2H), 6.77 (dd, *J* = 5.7, 2.4 Hz, 1H), 5.04–4.97 (m, 1H), 3.02 (dd, *J* = 15.2, 5.5 Hz, 1H), 2.70 (dd, *J* = 15.2, 8.3 Hz, 1H), 2.00–1.94 (m, 1H), 0.77 (d, *J* = 5.4 Hz, 4H); LCMS (ESI), m/z = 479.10 [M+1]^+^.

***N*-(4-(2,5-difluoro-4-(2-(2-oxoindolin-3-yl)acetamido)phenoxy)pyridin-2-yl)cyclopropanecarboxamide (6k)**

The amide coupling reaction of 2-(2-oxoindolin-3-yl)acetic acid (22.6 mg, 0.12 mmol) using DCC for 22 h via general procedure B was followed by NH_2_ silica gel chromatography (95% EtOAc/*n*-hexane) to afford 4 mg (8%) of *N*-(4-(2,5-difluoro-4-(2-(2-oxoindolin-3-yl)acetamido)phenoxy)pyridin-2-yl)cyclopropanecarboxamide as a white solid (98% purity): ^1^H NMR (400 MHz, DMSO-*d*_6_) *δ* 10.89 (s, 1H), 10.42 (s, 1H), 10.16 (s, 1H), 8.21 (d, *J* = 5.7 Hz, 1H), 8.07 (dd, *J* = 12.3, 7.2 Hz, 1H), 7.66 (d, *J* = 2.3 Hz, 1H), 7.53 (dd, *J* = 10.8, 7.4 Hz, 1H), 7.23–7.14 (m, 2H), 6.92 (t, *J* = 7.2 Hz, 1H), 6.83 (d, *J* = 7.6 Hz, 1H), 6.74 (dd, *J* = 5.7, 2.4 Hz, 1H), 3.81–3.76 (m, 1H), 3.11 (dd, *J* = 16.1, 4.9 Hz, 1H), 2.86 (dd, *J* = 16.1, 7.9 Hz, 1H), 1.97–1.94 (m, 1H), 0.81–0.74 (m, 4H); LCMS (ESI), m/z = 479.00 [M+1]^+^.

***N*-(4-(4-(2-(benzo[*d*]oxazol-2-yl)acetamido)-2,5-difluorophenoxy)pyridin-2-yl)cyclopropanecarboxamide (7a)**

The amide coupling reaction of 2-(benzo[*d*]oxazol-2-yl)acetic acid (17.4 mg, 0.10 mmol) using TBTU for 76 h via general procedure A was followed by NH_2_ silica gel chromatography (30%–50% EtOAc/*n*-hexane) to afford 8.5 mg (27%) of *N*-(4-(4-(2-(benzo[*d*]oxazol-2-yl)acetamido)-2,5-difluorophenoxy)pyridin-2-yl)cyclopropanecarboxamide as an ivory solid (98% purity): ^1^H NMR (400 MHz, DMSO-*d*_6_) *δ* 10.90 (s, 1H), 10.57 (s, 1H), 8.23 (d, *J* = 5.7 Hz, 1H), 8.14 (dd, *J* = 12.3, 7.2 Hz, 1H), 7.76–7.73 (m, 2H), 7.67 (d, *J* = 2.3 Hz, 1H), 7.59 (dd, *J* = 10.9, 7.4 Hz, 1H), 7.44–7.37 (m, 2H), 6.77 (dd, *J* = 5.7, 2.4 Hz, 1H), 4.31 (s, 2H), 2.01–1.91 (m, 1H), 0.86–0.77 (m, 4H); LCMS (ESI), m/z = 465.16 [M+1]^+^.

***N*-(4-(4-(2-(benzo[*d*]thiazol-2-yl)acetamido)-2,5-difluorophenoxy)pyridin-2-yl)cyclopropanecarboxamide (7b)**

The amide coupling reaction of 2-(benzo[*d*]thiazol-2-yl)acetic acid (30.4 mg, 0.16 mmol) using HATU for 30 h via general procedure A was followed by silica gel chromatography (45% EtOAc/*n*-hexane) to afford 5.7 mg (9%) of *N*-(4-(4-(2-(benzo[*d*]thiazol-2-yl)acetamido)-2,5-difluorophenoxy)pyridin-2-yl)cyclopropanecarboxamide as a yellow solid (98% purity): ^1^H NMR (400 MHz, acetone-*d*_6_) *δ* 10.12 (s, 1H), 9.80 (s, 1H), 8.40 (dd, *J* = 12.5, 7.3 Hz, 1H), 8.18 (d, *J* = 5.7 Hz, 1H), 8.08 (d, *J* = 7.9 Hz, 1H), 8.02 (d, *J* = 8.1 Hz, 1H), 7.87 (d, *J* = 2.3 Hz, 1H), 7.58–7.52 (m, 1H), 7.50–7.44 (m, 1H), 7.35 (dd, *J* = 10.9, 7.2 Hz, 1H), 6.70 (dd, *J* = 5.7, 2.4 Hz, 1H), 4.48 (s, 2H), 2.02–1.98 (m, 1H), 0.91–0.87 (m, 2H), 0.86–0.80 (m, 2H); LCMS (ESI), m/z = 481.20 [M+1]^+^.

***N*-(4-(4-(2-(1*H*-indol-2-yl)acetamido)-2,5-difluorophenoxy)pyridin-2-yl)cyclopropanecarboxamide (7c)**

The amide coupling reaction of 2-(1*H*-indol-2-yl)acetic acid (27.5 mg, 0.16 mmol) using HATU for 30 h via general procedure A was followed by NH_2_ silica gel chromatography (85% EtOAc/*n*-hexane) to afford 22.3 mg (36%) of *N*-(4-(4-(2-(1*H*-indol-2-yl)acetamido)-2,5-difluorophenoxy)pyridin-2-yl)cyclopropanecarboxamide as a pale brown solid (97% purity): ^1^H NMR (400 MHz, DMSO-*d*_6_) *δ* 11.00 (s, 1H), 10.90 (s, 1H), 10.25 (s, 1H), 8.21 (d, *J* = 5.7 Hz, 1H), 8.14 (dd, *J* = 12.4, 7.2 Hz, 1H), 7.65 (d, *J* = 2.3 Hz, 1H), 7.55 (dd, *J* = 10.9, 7.4 Hz, 1H), 7.45 (d, *J* = 7.8 Hz, 1H), 7.33 (d, *J* = 8.6 Hz, 1H), 7.05–7.00 (m, 1H), 6.97–6.91 (m, 1H), 6.75 (dd, *J* = 5.7, 2.4 Hz, 1H), 6.30 (s, 1H), 3.96 (s, 2H), 2.01–1.93 (m, 1H), 0.77 (d, *J* = 6.1 Hz, 4H); LCMS (ESI), m/z = 463.13 [M+1]^+^.

***N*-(4-(4-(3-(1*H*-indol-3-yl)propanamido)-2,5-difluorophenoxy)pyridin-2-yl)cyclopropanecarboxamide (7d)**

The amide coupling reaction of 2-(benzofuran-2-yl)acetic acid (27.7 mg, 0.16 mmol) using HATU for 30 h via general procedure A was followed by NH_2_ silica gel chromatography (35% EtOAc/*n*-hexane) to afford 6.5 mg (11%) of *N*-(4-(4-(2-(benzofuran-2-yl)acetamido)-2,5-difluorophenoxy)pyridin-2-yl)cyclopropanecarboxamide as a pale yellow solid (99% purity): ^1^H NMR (400 MHz, DMSO-*d*_6_) *δ* 10.90 (s, 1H), 10.38 (s, 1H), 8.22 (d, *J* = 5.7 Hz, 1H), 8.12 (dd, *J* = 12.3, 7.2 Hz, 1H), 7.67 (d, *J* = 2.2 Hz, 1H), 7.62–7.52 (m, 3H), 7.30–7.20 (m, 2H), 6.81 (s, 1H), 6.76 (dd, *J* = 5.7, 2.4 Hz, 1H), 4.09 (s, 2H), 2.00–1.96 (m, 1H), 0.81–0.74 (m, 4H); LCMS (ESI), m/z = 464.00 [M+1]^+^.

***N*-(4-(4-(2-(benzo[*b*]thiophen-2-yl)acetamido)-2,5-difluorophenoxy)pyridin-2-yl)cyclopropanecarboxamide (7e)**

The amide coupling reaction of 2-(benzo[*b*]thiophen-2-yl)acetic acid (30.2 mg, 0.16 mmol) using HATU for 21 h via general procedure A was followed by NH_2_ silica gel chromatography (35% EtOAc/*n*-hexane) to afford 5.5 mg (8%) of *N*-(4-(4-(2-(benzo[*b*]thiophen-2-yl)acetamido)-2,5-difluorophenoxy)pyridin-2-yl)cyclopropanecarboxamide as a yellow solid (96% purity): ^1^H NMR (400 MHz, acetone-*d*_6_) *δ* 9.78 (s, 1H), 9.52 (s, 1H), 8.37 (dd, *J* = 12.6, 7.3 Hz, 1H), 8.17 (d, *J* = 5.7 Hz, 1H), 7.87 (dd, *J* = 13.7, 4.8 Hz, 2H), 7.83–7.76 (m, 1H), 7.40–7.27 (m, 4H), 6.69 (dd, *J* = 5.7, 2.4 Hz, 1H), 4.22 (s, 2H), 1.98–1.97 (m, 1H), 0.91–0.87 (m, 2H), 0.85–0.80 (m, 2H); LCMS (ESI), m/z = 480.00 [M+1]^+^.

***N*-(4-(4-(3-(benzo[*d*]oxazol-2-yl)propanamido)-2,5-difluorophenoxy) pyridin-2-yl)cyclopropanecarboxamide (7f)**

The amide coupling reaction of 3-(benzo[*d*]oxazol-2-yl)propanoic acid (30.1 mg, 0.16 mmol) using HATU for 17 h via general procedure A was followed by NH_2_ silica gel chromatography (45% EtOAc/*n*-hexane) to afford 38.4 mg (60%) of *N*-(4-(4-(3-(benzo[*d*]oxazol-2-yl)propanamido)-2,5-difluorophenoxy)pyridin-2-yl)cyclopropanecarboxamide as a pale yellow solid (98% purity): ^1^H NMR (400 MHz, DMSO-*d*_6_) *δ* 10.90 (s, 1H), 10.21 (s, 1H), 8.21 (d, *J* = 5.7 Hz, 1H), 8.09 (dd, *J* = 12.4, 7.2 Hz, 1H), 7.69 – 7.66 (m, 2H), 7.65 (d, *J* = 2.4 Hz, 1H), 7.53 (dd, *J* = 10.9, 7.4 Hz, 1H), 7.39–7.30 (m, 2H), 6.74 (dd, *J* = 5.7, 2.4 Hz, 1H), 3.26 (t, *J* = 6.9 Hz, 2H), 3.08–3.05 (m, 2H), 2.01–1.93 (m, 1H), 0.77 (d, *J* = 5.7 Hz, 4H); LCMS (ESI), m/z = 479.13 [M+1]^+^.

***N*-(4-((2-(cyclopropanecarboxamido)pyridin-4-yl)oxy)-2,5-difluorophenyl)-1*H*-benzo[*d*]imidazole-6-carboxamide (8a)**

The amide coupling reaction of 1*H*-benzo[*d*]imidazole-6-carboxylic acid (25.5 mg, 0.16 mmol) using DCC for 18 h via general procedure B was followed by silica gel chromatography (5% MeOH/CH_2_Cl_2_) to afford 3.8 mg (6%) of *N*-(4-((2-(cyclopropanecarboxamido)pyridin-4-yl)oxy)-2,5-difluorophenyl)-1*H*-benzo[*d*]imidazole-6-carboxamide as a white solid (96% purity): ^1^H NMR (400 MHz, DMSO-*d*_6_) *δ* 12.80 (s, 1H), 10.93 (s, 1H), 10.30 (s, 1H), 8.40 (s, 1H), 8.32 (s, 1H), 8.25 (d, *J* = 5.7 Hz, 1H), 7.90–7.83 (m, 2H), 7.74–7.68 (m, 2H), 7.58 (dd, *J* = 10.3, 7.4 Hz, 1H), 6.80 (dd, *J* = 5.7, 2.4 Hz, 1H), 2.01–1.97 (m, 1H), 0.82–0.76 (m, 4H); LCMS (ESI), m/z = 450.00 [M+1]^+^.

***N*-(4-((2-(cyclopropanecarboxamido)pyridin-4-yl)oxy)-2,5-difluorophenyl)benzo[*d*]oxazole-6-carboxamide (8b)**

The amide coupling reaction of benzo[*d*]oxazole-6-carboxylic acid (25.6 mg, 0.16 mmol) using HATU for 30 h via general procedure A was followed by silica gel chromatography (50% EtOAc/*n*-hexane) to afford 27.8 mg (47%) of *N*-(4-((2-(cyclopropanecarboxamido)pyridin-4-yl)oxy)-2,5-difluorophenyl)benzo[*d*]oxazole-6-carboxamide as a pale yellow solid (99% purity): ^1^H NMR (400 MHz, DMSO-*d*_6_) *δ* 10.95 (s, 1H), 10.47 (s, 1H), 8.96 (s, 1H), 8.41 (d, *J* = 1.1 Hz, 1H), 8.25 (d, *J* = 5.7 Hz, 1H), 8.06 (dd, *J* = 8.4, 1.5 Hz, 1H), 7.98 (d, *J* = 8.3 Hz, 1H), 7.87 (dd, *J* = 11.5, 7.1 Hz, 1H), 7.70 (d, *J* = 2.3 Hz, 1H), 7.62 (dd, *J* = 10.3, 7.4 Hz, 1H), 6.81 (dd, *J* = 5.7, 2.4 Hz, 1H), 2.02–1.95 (m, 1H), 0.79 (d, *J* = 6.2 Hz, 4H); LCMS (ESI), m/z = 451.16 [M+1]^+^.

***N*-(4-((2-(cyclopropanecarboxamido)pyridin-4-yl)oxy)-2,5-difluorophenyl)benzo[*d*]thiazole-6-carboxamide (8c)**

The amide coupling reaction of benzo[*d*]thiazole-6-carboxylic acid (28.2 mg, 0.16 mmol) using HATU for 21 h via general procedure A was followed by NH_2_ silica gel chromatography (35% EtOAc/*n*-hexane) to afford 6.2 mg (10%) of *N*-(4-((2-(cyclopropanecarboxamido)pyridin-4-yl)oxy)-2,5-difluorophenyl)benzo[*d*]thiazole-6-carboxamide as a yellow solid (96% purity): ^1^H NMR (400 MHz, DMSO-*d*_6_) *δ* 10.94 (s, 1H), 10.50 (s, 1H), 9.59 (s, 1H), 8.83 (d, *J* = 1.4 Hz, 1H), 8.25–8.23 (m, 2H), 8.13 (dd, *J* = 8.6, 1.7 Hz, 1H), 7.90 (dd, *J* = 11.6, 7.1 Hz, 1H), 7.70 (d, *J* = 2.3 Hz, 1H), 7.60 (dd, *J* = 10.3, 7.4 Hz, 1H), 6.80 (dd, *J* = 5.7, 2.4 Hz, 1H), 2.02–1.94 (m, 1H), 0.78 (d, *J* = 6.1 Hz, 4H); LCMS (ESI), m/z = 467.06 [M+1]^+^.

***N*-(4-((2-(cyclopropanecarboxamido)pyridin-4-yl)oxy)-2,5-difluorophenyl)benzofuran-6-carboxamide (8e)**

The amide coupling reaction of benzofuran-6-carboxylic acid (25.5 mg, 0.16 mmol) using HATU for 18 h via general procedure A was followed by silica gel chromatography (35% EtOAc/*n*-hexane) to afford 8 mg (13%) of *N*-(4-((2-(cyclopropanecarboxamido)pyridin-4-yl)oxy)-2,5-difluorophenyl)benzofuran-6-carboxamide as a white solid (95% purity): ^1^H NMR (400 MHz, DMSO-*d*_6_) *δ* 10.93 (s, 1H), 10.34 (s, 1H), 8.29 – 8.23 (m, 2H), 8.21 (d, *J* = 2.2 Hz, 1H), 7.94–7.81 (m, 3H), 7.71 (d, *J* = 2.3 Hz, 1H), 7.59 (dd, *J* = 10.3, 7.4 Hz, 1H), 7.10 (dd, *J* = 2.1, 0.8 Hz, 1H), 6.80 (dd, *J* = 5.7, 2.4 Hz, 1H), 2.03–1.95 (m, 1H), 0.79 (d, *J* = 5.8 Hz, 4H); LCMS (ESI), m/z = 450.00 [M+1]^+^.

***N*-(4-((2-(cyclopropanecarboxamido)pyridin-4-yl)oxy)-2,5-difluorophenyl)benzo[*b*]thiophene-6-carboxamide (8f)**

The amide coupling reaction of benzo[*b*]thiophene-6-carboxylic acid (28.0 mg, 0.16 mmol) using HATU for 30 h via general procedure A was followed by NH_2_ silica gel chromatography (20% EtOAc/*n*-hexane) to afford 4.5 mg (7%) of *N*-(4-((2-(cyclopropanecarboxamido)pyridin-4-yl)oxy)-2,5-difluorophenyl)benzo[*b*]thiophene-6-carboxamide as a white solid (95% purity): ^1^H NMR (400 MHz, DMSO-*d*_6_) *δ* 10.93 (s, 1H), 10.39 (s, 1H), 8.68 (s, 1H), 8.25 (d, *J* = 5.7 Hz, 1H), 8.06–7.97 (m, 3H), 7.89 (dd, *J* = 11.7, 7.0 Hz, 1H), 7.71 (d, *J* = 2.3 Hz, 1H), 7.62–7.56 (m, 2H), 6.80 (dd, *J* = 5.7, 2.4 Hz, 1H), 2.00–1.96 (m, 1H), 0.79 (d, *J* = 5.6 Hz, 4H); LCMS (ESI), m/z = 466.00 [M+1]^+^.

***N*-(4-((2-(cyclopropanecarboxamido)pyridin-4-yl)oxy)-2,5-difluorophenyl)-1*H*-indole-6-carboxamide (8g)**

The amide coupling reaction of 1-(*tert*-butoxycarbonyl)-1*H*-indole-6-carboxylic acid (82.0 mg, 0.31 mmol) using DCC for 72 h via general procedure B was followed by prep-HPLC (20%–100% water/acetonitrile with 0.1% TFA) to afford 10.5 mg (7%) of *tert*-butyl 6-((4-((2-(cyclopropanecarboxamido)pyridin-4-yl)oxy)-2,5-difluorophenyl)carbamoyl)-1*H*-indole-1-carboxylate (**8d**) as a mixture. A solution of *tert*-butyl 6-((4-((2-(cyclopropanecarboxamido)pyridin-4-yl)oxy)-2,5-difluorophenyl)carbamoyl)-1*H*-indole-1-carboxylate mixture (10.5 mg) in CH_2_Cl_2_ (736.0 μL) was added with 4M HCl in 1,4-dioxane (190 µl, 0.76 mmol) at room temperature, and the mixture was stirred for 19 h. The solvent was evaporated under reduced pressure. The residue was purified by NH_2_ silica gel chromatography (100% EtOAc/n-hexane) to afford 2.9 mg (2%, for 2 steps) of *N*-(4-((2-(cyclopropanecarboxamido)pyridin-4-yl)oxy)-2,5-difluorophenyl)-1*H*-indole-6-carboxamide as a beige solid (98% purity): ^1^H NMR (400 MHz, acetone-*d*_6_) *δ* 10.73 (s, 1H), 9.83 (s, 1H), 9.31 (s, 1H), 8.32 (dd, *J* = 12.4, 7.2 Hz, 1H), 8.23–8.15 (m, 2H), 7.89 (d, *J* = 2.4 Hz, 1H), 7.76–7.67 (m, 2H), 7.57 (t, *J* = 2.8 Hz, 1H), 7.36 (dd, *J* = 10.7, 7.3 Hz, 1H), 6.72 (dd, *J* = 5.7, 2.4 Hz, 1H), 6.61–6.57 (m, 1H), 1.98–1.94 (m, 1H), 0.91–0.86 (m, 2H), 0.86–0.79 (m, 2H); LCMS (ESI), m/z = 490.45 [M+ ACN +1]^+^.

***N*-(4-((2-(cyclopropanecarboxamido)pyridin-4-yl)oxy)-2,5-difluorophenyl) -2-oxo-1,2,3,4-tetrahydroquinoline-3-carboxamide (9a)**

The amide coupling reaction of 2-oxo-1,2,3,4-tetrahydroquinoline-3-carboxylic acid (18.8 mg, 0.1 mmol) using TBTU for 19 h via general procedure A was followed by prep-HPLC (20%–100% water/acetonitrile with 0.1% TFA) to afford 24.0 mg (61%) of *N*-(4-((2-(cyclopropanecarboxamido)pyridin-4-yl)oxy)-2,5-difluorophenyl)-2-oxo-1,2,3,4-tetrahydroquinoline-3-carboxamide as a pale yellow solid (99% purity): ^1^H NMR (400 MHz, DMSO-*d*_6_) *δ* 10.94 (s, 1H), 10.49 (s, 1H), 10.37 (s, 1H), 8.25–8.20 (m, 2H), 7.65 (d, *J* = 2.2 Hz, 1H), 7.58 (dd, *J* = 10.9, 7.4 Hz, 1H), 7.25–7.17 (m, 2H), 6.97 (t, *J* = 7.3 Hz, 1H), 6.90 (d, *J* = 7.8 Hz, 1H), 6.78 (dd, *J* = 5.7, 2.4 Hz, 1H), 3.92 (dd, *J* = 12.1, 6.6 Hz, 1H), 3.24 (dd, *J* = 15.6, 12.5 Hz, 1H), 3.13 (dd, *J* = 16.0, 6.6 Hz, 1H), 2.01–1.95 (m, 1H), 0.83–0.76 (m, 4H); LCMS (ESI), m/z = 479.12 [M+1]^+^.

***N*-(4-((2-(cyclopropanecarboxamido)pyridin-4-yl)oxy)-2,5-difluorophenyl) -1-oxo-1,2,3,4-tetrahydroisoquinoline-3-carboxamide (9b)**

The amide coupling reaction of 1-oxo-1,2,3,4-tetrahydroisoquinoline-3-carboxylic acid (18.8mg, 0.1 mmol) using TBTU for 88 h via general procedure A was followed by prep-HPLC (20%–100% water/acetonitrile with 0.1% TFA) to afford 2.6 mg (7%) *N*-(4-((2-(cyclopropanecarboxamido)pyridin-4-yl)oxy)-2,5-difluorophenyl)-1-oxo-1,2,3,4-tetrahydroisoquinoline-3-carboxamide as a pale yellow solid (97% purity): ^1^H NMR (400 MHz, CD_3_OD) *δ* 8.24 (d, *J* = 6.6 Hz, 1H), 8.08 (dd, *J* = 12.0, 7.1 Hz, 1H), 8.00 (dd, *J* = 7.7, 0.9 Hz, 1H), 7.53 (td, *J* = 7.5, 1.3 Hz, 1H), 7.42–7.30 (m, 3H), 7.12 (d, *J* = 2.2 Hz, 1H), 7.02 (dd, *J* = 6.6, 2.5 Hz, 1H), 4.62 (t, *J* = 5.9 Hz, 1H), 3.51 (dd, *J* = 16.2, 6.1 Hz, 1H), 3.38–3.34 (m, 1H), 1.87–1.81 (m, 1H), 1.10–0.99 (m, 4H); LCMS (ESI), m/z = 479.10 [M+1]^+^.

***N*-(4-((2-(cyclopropanecarboxamido)pyridin-4-yl)oxy)-2,5-difluorophenyl)-1*H*-indene-3-carboxamide (9c)**

The amide coupling reaction of 1*H*-indene-3-carboxylic acid (25.2 mg, 0.16 mmol) using HATU for 50 h via general procedure A was followed by silica gel chromatography (50% EtOAc/*n*-hexane) to afford 5.2 mg (9%) of *N*-(4-((2-(cyclopropanecarboxamido)pyridin-4-yl)oxy)-2,5-difluorophenyl)-1*H*-indene-3-carboxamide as a pale yellow solid (96% purity): ^1^H NMR (400 MHz, DMSO-*d*_6_) *δ* 10.95 (s, 1H), 10.23 (s, 1H), 8.24 (d, *J* = 5.7 Hz, 1H), 7.94 (d, *J* = 7.4 Hz, 1H), 7.89 (dd, *J* = 11.7, 7.1 Hz, 1H), 7.69 (d, *J* = 2.3 Hz, 1H), 7.62–7.51 (m, 3H), 7.33 (t, *J* = 7.0 Hz, 1H), 7.27 (td, *J* = 7.4, 1.1 Hz, 1H), 6.80 (dd, *J* = 5.7, 2.4 Hz, 1H), 3.66 (s, 2H), 1.99–1.96 (m, 1H), 0.78 (d, *J* = 6.1 Hz, 4H); LCMS (ESI), m/z = 448.13 [M+1]^+^.

**Chemicals**

Forskolin (FSK) purchased from Tocris Bioscience (Bristol, UK) was used at a concentration of 10 μM unless otherwise specified. Sorafenib, foretinib, cabozantinib, and ALT were purchased from Medchem express (Monmouth Junction, NJ, USA) and were used as indicated in the figure legends.

**Cell culture**

Mel-Ab mouse melanocytes, as described previously, were maintained in Dulbecco’s modified Eagle’s medium (DMEM, Welgene, Gyeongsan-si, Korea) supplemented with 10% fetal bovine serum (FBS, Life Sciences, Corning, NY, USA), 100 nM 12-O-tetradecanoylphorbol-13-acetate (Sigma-Aldrich), 1 nM cholera toxin (Cayman Chemicals, Ann Arbor, MI, USA), and 1% antibiotic–antimycotic solution (AA, Thermo Fisher Scientific, Waltham, MA, USA). 293T cells and B16F10 melanoma cells were purchased from the Korean Cell Line Bank (Seoul, Korea) and were maintained in DMEM (Welgene) supplemented with 10% FBS (Life Sciences) and 1% AA (Thermo Fisher Scientific). Primary NHMs obtained from Invitrogen (Carlsbad, CA, USA) were maintained in Medium 254 (Invitrogen) supplemented with Human Melanocyte Growth Supplement (HMGS, Invitrogen) and 1% A.A. (Thermo Fisher Scientific). All cells were maintained in a humid environment with 5% CO_2_ at 37°C. Primary mouse melanocytes were isolated from neonatal KRT14-SCF mice. Briefly, after the euthanasia of the neonatal KRT14-SCF mice, the entire skin tissue was separated. After washing with phosphate-buffered saline (PBS), the separated skin tissue was incubated in PBS containing 5 mg/mL Dispase II (Sigma-Aldrich) at 37 °C for 2 h, washed with PBS, and then cultured in DMEM supplemented with 10% FBS, 100 nM 12-O-tetradecanoylphorbol-13-acetate, 1 nM cholera toxin, and 1% AA.

**Melanin content and tyrosinase activity**

Mel-Ab cells, primary mouse melanocytes, and B16F10 cells plated in DMEM supplemented with 10% FBS and 1% AA or NHM plated in Medium 254 supplemented with HMGS and 1% AA were treated with vehicle or chemicals in the absence or presence of FSK, as indicated in the figure legends. In cases of co-treatment with FSK, pretreatment with specific drugs started 30 min before FSK. At 72 h of treatment, cells were solubilized with 1 N NaOH, boiled for 30 min with intermittent vortexing, and then centrifuged at 13,000 rpm. The melanin content in the supernatant was measured at an optical density of 405 nm using a microplate reader (Biotek, Winooski, VT, USA). The obtained results were normalized to the total protein amount of the lysate, and the melanin content was expressed as a percent change relative to the vehicle-treated controls. The level of cellular tyrosinase activity was evaluated by measuring the rate of dopachrome formation from L-3,4-dihydroxyphenylalanine (L-DOPA). The cellular lysates from Mel-ab, primary mouse melanocytes, B16F10, and NHM cells under the same conditions as in the melanin content experiments were prepared in a tyrosinase lysis buffer (phosphate buffer, pH 6.8 containing 1% Triton X-100), and tyrosinase activity was measured as previously described. Moreover, 1 µM of ALT and ALT analogs were used for in vitro direct tyrosinase activity using mushroom tyrosinase following the instruction of the manufacturer (Sigma-Aldrich). Then, 100 ng/mL arbutin or 200 μM kojic acid treatment was used as a positive control, and tyrosinase activity was normalized to the lysate total protein and expressed as a percent change relative to the vehicle-treated controls.

**Cell viability**

Mel-Ab cells, primary mouse melanocytes, and B16F10 cells plated in DMEM supplemented with 10% FBS and 1% AA or NHM plated in Medium 254 supplemented with HMGS and 1% AA were treated with vehicle or chemicals. At 72 h after treatment, cell viability was assessed by the MTT assay (Duchefa-biochemie, Haarlem, Netherlands) according to the manufacturer’s instructions. The obtained results were presented as the percent change relative to the vehicle-treated controls.

**mRNA expression analysis**

Total RNA was isolated using the FavorPrep Blood/Cultured Cell Total RNA Purification Kit following the manufacturer’s instructions (Farvorgen Biotech, Changzhi Township, Taiwan). Then, a 1-μg sample was used for the first-strand cDNA synthesis with the ReverTra Ace qPCR RT Kit (Toyobo, Osaka, Japan) and random hexamer according to the manufacturer’s instructions. The relative expression levels of the target mRNAs were compared by quantitative real-time reverse–transcriptase polymerase chain reaction using the Revert Aid First Strand cDNA Synthesis Kit (Thermo Fisher Scientific) and Lightcycler 480II (Roche Applied Science, Indianapolis, IN, USA). The expression of GAPDH was used as an internal reference. Specific primer sets for amplifying the target genes are listed in Table S1.

**Antibodies and immunoblotting**

Protein samples from cultured cells were prepared in lysis buffer containing 10 mM Tris, 5 mM EDTA, and 1% SDS (pH 7.4). Protein samples from mouse or human skin tissues were prepared by first grinding the tissue in liquid nitrogen before lysis in a buffer containing 20 mM HEPES, 150 mM NaCl, 1 mM EDTA, 1 mM EGTA, 1% Triton X-100, protease inhibitor cocktail (Tech & Innovation, ChunCheon, Korea), and phosphatase inhibitors (5 mM Na-pyrophosphate, 20 mM β-glycerophosphate, and 50 mM NaF). Approximately 20 μg of protein were separated by 5.5%–8% sodium dodecyl-sulfate polyacrylamide gel electrophoresis, transferred to 0.45 μm nitrocellulose membranes (GE Healthcare, Chicago, IL, USA), blocked with 3% bovine serum albumin (BSA, Bovogen Biologicals, Keilor East, Australia) in Tris-buffered saline containing 0.1% Tween 20, and subjected to immunoblotting. Protein bands were visualized using ECL Western blotting detection reagents (Thermo Fisher Scientific) exposed to Medical X-ray Film (AGFA, Mortsel, Belgium) or Chemidoc^TM^ MP (Thermo Fisher Scientific). Antibodies against MITF, tyrosinase, TYRP1, and DCT were purchased from Abcam (Cambridge, UK). AKT, phospho-AKT, AMPK, phospho-AMPK, CREB, phospho-CREB, ERK, phospho-ERK, JNK, phospho-JNK, phospho-PKA substrate, and phospho-PKC subsubstrate were purchased from Cell Signaling Technology (Danvers, MA, USA), and MLANA antibody was obtained from Cell Marque (Rocklin, CA, USA). The CRTC3 antibody produced in-house or by Cell Signaling Technology was used for immunoblotting, and the CRTC3 antibody acquired from Abcam was used for immunofluorescence (IF) experiments. HSP90 (Dallas, TX, USA) or α-tubulin (Gentex, Holland, MI, USA) was used as internal loading controls for immunoblotting.

**CRTC3 activity**

The promoter-based reporter assay was employed to assess the transcriptional activity of CRTC3. Briefly, a CRTC3/CREB reporter cell line was established by introducing a fusion plasmid containing human *Evx1* (220 bp) promoters and gaussia luciferase into HEK-293T cells. The CRTC3/CREB reporter cells were subjected to treatments as indicated in the figures. After 24 h of treatment, the Gaussia luciferase activity was measured as previously described, and the resulting data were presented as the percentage change relative to the vehicle-treated controls.

**Subcellular localization of CRTC3**

To examine the subcellular localization of CRTC3 in B16F10 cells, plasmid constructs encoding the *CRTC3–EGFP* fusion gene were transfected into B16F10 cells using PEI (Sigma-Aldrich) or lipofectamine reagents (Invitrogen). Between 24 h and 48 h after transfection, *CRTC3–EGFP*-transfected B16F10 cells were treated with the vehicle (DMSO), 1 µM ALT, or ALT analogs (ALT6a or ALT7a), in the presence or absence of 10 µM FSK for 1 h, and subcellular localization of CRTC3 was monitored by fluorescence microscopy (Observer.Z1, Carl Zeiss, Oberkochen, Germany). Mel-ab cells infected with the lentivirus containing *CRTC3-GFP* were then treated with the vehicle (DMSO), 1 µM ALT, ALT6a, or ALT7a in the presence or absence of 10 µM FSK for 1 h and were subjected to fluorescence microscopy using an Observer.Z1 microscope (Carl Zeiss). For cell fractionation experiments, Mel-ab cells were treated with the vehicle (DMSO), 1 µM ALT, ALT6a, or ALT7a in the presence or absence of FSK for 1 h, and cell fractionation was performed using the NE-PER Nuclear and Cytoplasmic Extraction Kit (Thermo Fisher Scientific) according to the manufacturer’s instructions.

**Skin PMAPA**

Reagents for PAMPA were obtained from Pion, Inc. (MA, USA), and experiments were conducted by following the manufacturer’s instructions. Briefly, the pre-coated upper chamber of the SKIN PAMPA sandwich plate was incubated with a hydration solution for 12–18 h at room temperature a day before the experiment. Then, 200 μL of the 25 μM test compound in Prisma HT buffer was added to the lower chamber of the PAMPA sandwich plate. Moreover, 200 μL of the same Prisma HT buffer was added to the hydrated upper chamber of the sandwich plate and was combined with the lower chamber. Then, the plate was kept at room temperature for 5 h, and solutions from each compartment were measured with UV absorbance between 250 nm and 500 nm using a microplate reader (Synergy H1, Biotek). The P_e_ value was calculated using PAMPA Explorer version 3.8 (Pion).

**Animals**

All animals used in this study were subjected to the protocols approved by the Institutional Animal Care and Use Committee of the Asan Medical Center, Seoul, Korea (2020-02-248). KRT14-SCF transgene-harbored C57BL6/J mice (KRT14-SCF+/-), obtained from Jackson Laboratories (Bar Harbor, USA), were bred with C57BL6/J WT mice, resulting in KRT14-SCF+/- (K14-SCF) or KRT14-SCF-/- (WT), as described previously. All mice were raised under controlled conditions, including specific pathogen-free environments, 12-h light/dark cycle, free access to water, and normal chow diet (Purina, Pyeongtaek, Republic of Korea). For topical application of drugs, 5 or 10 μM ALT6a or ALT7a was prepared in a standard dermatological vehicle of 70% ethanol and 30% propylene glycol (Sigma-Aldrich). Thereafter, 100 µL of the prepared agent was applied daily to the tails of 8-week-old C57BL6/J mice or KRT14-SCF mice and spread evenly over the tail with gloved fingers. The mice were sacrificed 35 days after the first treatment, and tissue sections and protein samples were prepared.

**Ex vivo human skin tissue culture**

Human skin tissue was acquired from consenting patients (IRB no. 2020-0091) who received neck or abdomen reduction surgery. Skin tissues were briefly washed with 100% EtOH, followed by 70% EtOH, cut into approximately 2 cm × 2 cm sections, and placed on metal grids in 6-well plates in contact with DMEM containing 5% FBS and 10% AA under a humidified environment of 5% CO_2_ at 37 °C. The culture medium was replaced every day. For UVR-stimulated melanogenesis, the skin tissue was exposed to 200 mJ UVB for 30 s every 48 h with the vehicle, ALT, ALT6a, or ALT7a topically in a standard dermatological vehicle of 70% ethanol and 30% propylene glycol (Sigma-Aldrich). After 24 h or 96 h, skin tissues were harvested, divided into two parts: one part was embedded in paraffin for histology, Fontana–Masson, and immunofluorescence (IF) staining, and the other part was ground in liquid nitrogen for the isolation of protein samples as described above.

**Fontana–Masson staining, melanin index, and immunofluorescence staining**

Paraffin-embedded human skin tissues were cut into 4-μm thick sections and subjected to Fontana–Masson staining following the instructions of the manufacturer (ID Laboratories, London, ON, Canada). Multiple random areas were photographed using a phase-contrast microscope (BX53, Olympus, Tokyo, Japan), and the melanin index was calculated by measuring the stained area relative to the total epidermal area using Image J (National Institute of Health, Bethesda, MA, USA). The results were presented as a percent change relative to the vehicle-treated controls. For immunohistochemistry, paraffin-embedded 5-μm thick human skin sections were mounted on slides and rehydrated in descending concentrations of EtOH. After washing with TBST, the antigen was retrieved by incubating slides in a streamer containing 10 mM citrate buffer for 20 min. Then, the slides were incubated in 3% H_2_O_2_ for 10 min, followed by 3N HCl for 30 min. After washing with TBST, the slides were incubated with 3% BSA in TBST for 1 h and then with primary antibodies (MLANA and CRTC3). The FITC-conjugated anti-mouse secondary antibody was used to detect CRTC3, and anti-rabbit Alexa Fluor 546 was used to detect MLANA at 4 °C for 30 min. Images were acquired using a Zeiss LSM 780 laser scanning confocal microscope (Leica; Wetzlar, Germany).

**Statistics**

Data are presented as means ± standard errors of the mean (s.e.m). The means were compared between the treatment groups using an unpaired Student’s t-test, utilizing GraphPad Prism version 5. Statistical significance was denoted by *, **, and *** or ^+^, ^++^, and ^+++^ for p < 0.5, p < 0.1, and p < 0.01, respectively (two-tailed), and were considered significant for all tests.

**Supplementary Table**

| Name | Forward | Reverse |
| --- | --- | --- |
| GAPDH | CATCACTGCCACCCAGAAGACTG | ATGCCAGTGAGTTCCCGTTCAG |
| MITF | GGGATGCCTTGTTTATGGTG | CACCGCAGACCACTTAGTCC |
| Tyrosinase | TTATGCGATGGAACACCTGA | GAGCGGTATGAAAGGAACCA |
| Tyrp1 | CCCCTAGCCTATATCTCCCT | TACCATCGTGGGGATAATGG |
| DCT | CTTTGCAACCGGGAAGAACG | CCGACTAATCAGCGTTGGGT |

**Table S1.** List of primers used for the quantitative real-time reverse-transcription polymerase chain reaction.

**Supplementary figures**

**
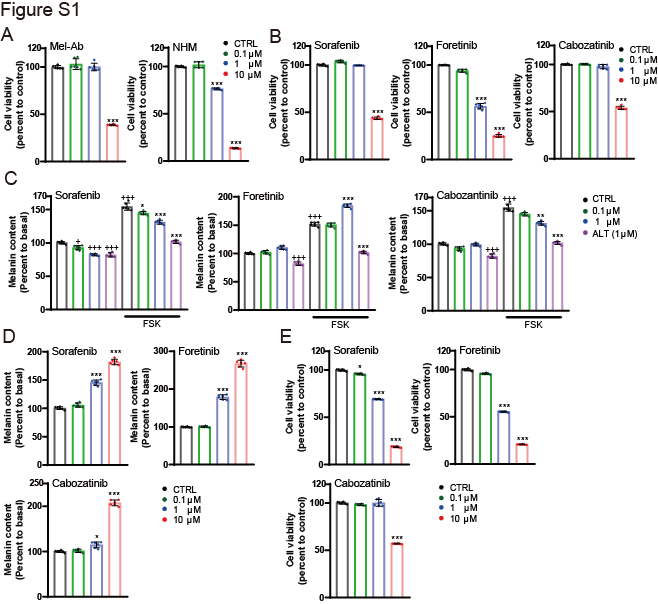
**

**Fig. S1. Effect of functional analogs of altiratinib on cell viability and melanogenesis.** (A) Cell viability of Mel-Ab and NHM after 72-h treatment with 0.1–10 μM altiratinib. (B) Cell viability assessed by the MTT assay and (C) melanin content in mouse melanocytes (Mel-Ab) under basal or FSK-stimulated conditions after 72-h treatment with 0.1–10 μM sorafenib, foretinib, and cabozantinib. (D) Melanin content and (E) cell viability in normal human melanocytes (NHM) after 72-h treatment with 0.1–10 μM sorafenib, foretinib, and cabozantinib.


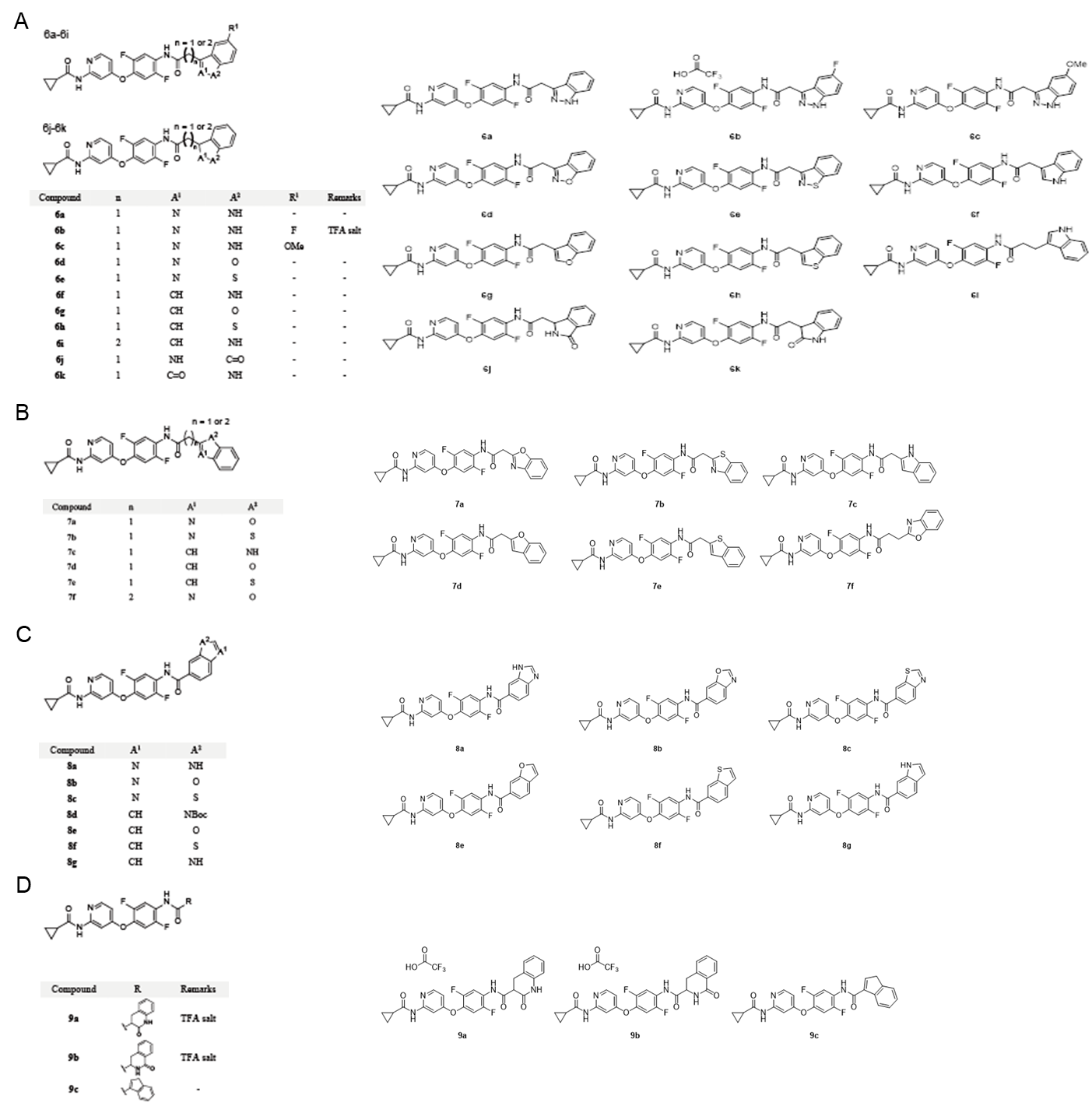


**Fig.S2. Strategy for the design and chemical structure of ALT analogs series (A) 6, (B) 7, (C) 8, and (D) 9.**


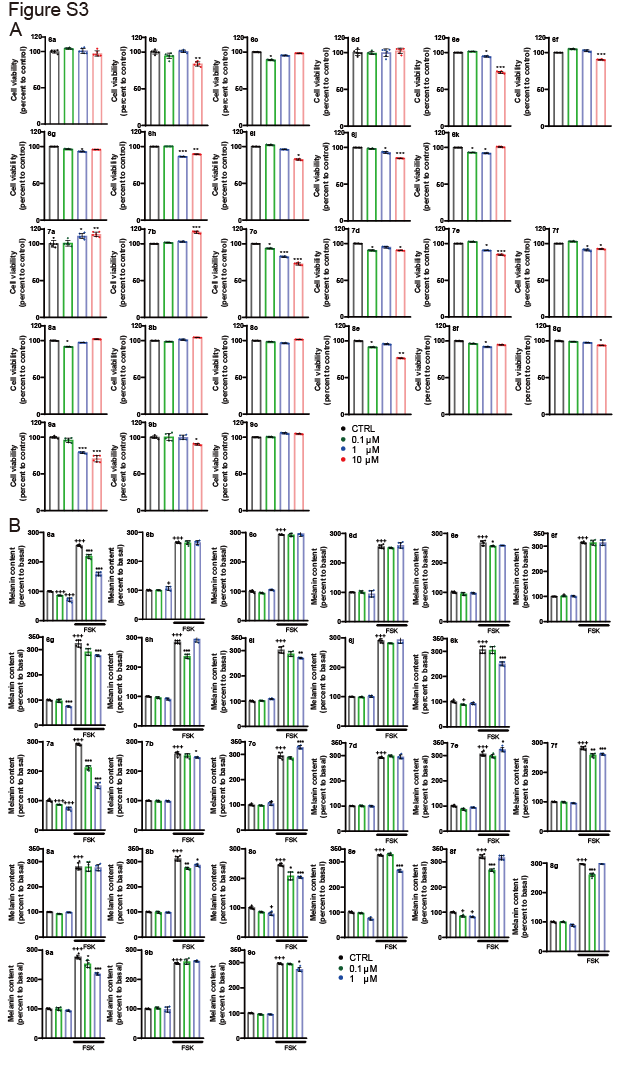


**Fig. S3. Effect of altiratinib analogs on cell viability and melanogenesis of normal mouse melanocytes.** (A) Mel-Ab cell viability assessed by the MTT assay after 72 h of treatment with 0.1–10 μM ALT analog series 6, 7, 8, and 9. (B) Melanin content in Mel-Ab cells for 72 h under basal and FSK-stimulation conditions after treatment with 0.1 and 1 μM altiratinib analog series 6, 7, 8, and 9. Data are displayed as percent change from the vehicle-treated controls (CTRL).


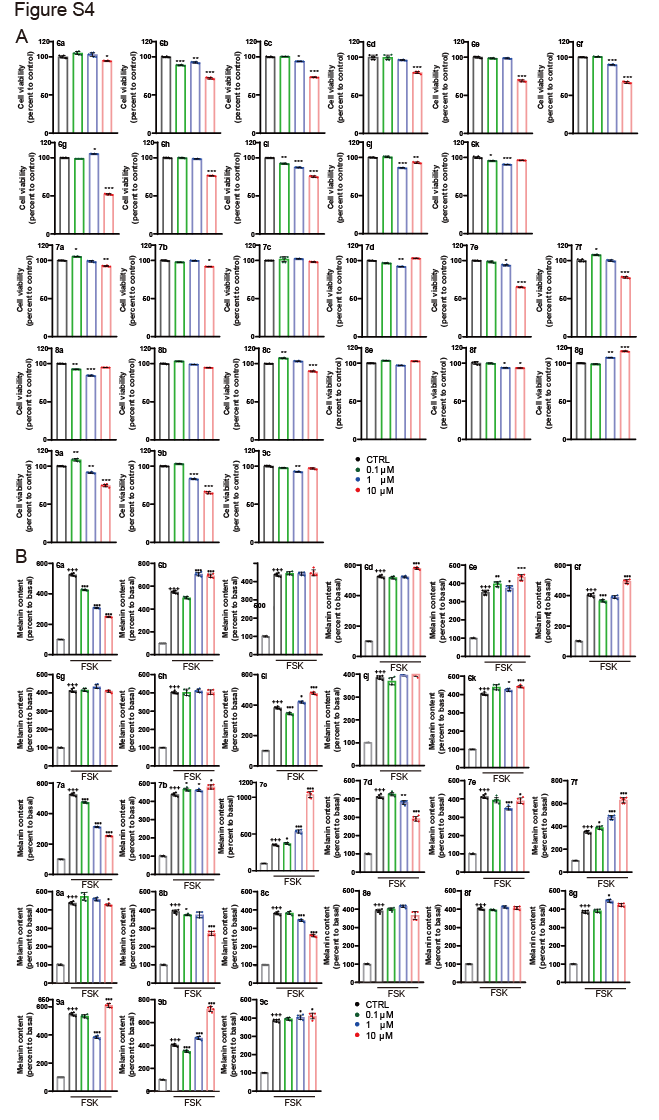


**Fig. S4. Effect of altiratinib analogs on cell viability and melanogenesis of B16F10 melanoma cells.** (A) Cellular viability of B16F10 melanoma cells after 72 h of treatment with 0.1–10 μM altiratinib analog series 6, 7, 8, and 9 assessed by the MTT assay. (B) Melanin content in B16F10 melanoma cells for 72 h under basal and FSK-stimulation conditions after treatment with 0.1–10 μM altiratinib analog series 6, 7, 8, and 9. Data are displayed as percent change from the vehicle-treated controls (CTRL).


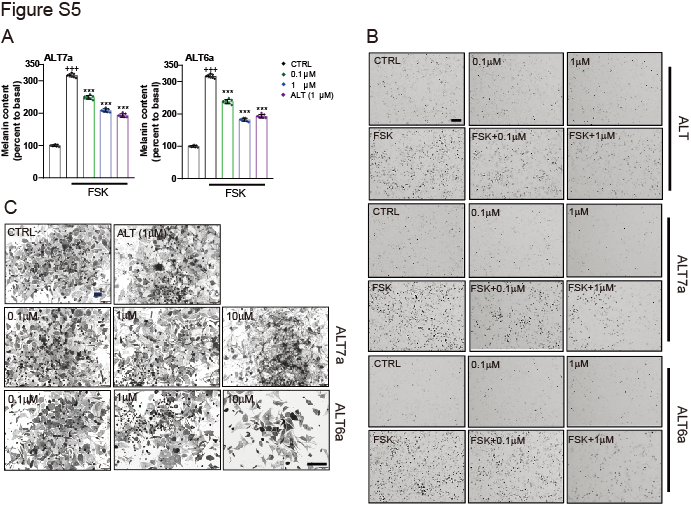


**Fig. S5.** **Decreased melanin production in mouse melanocytes.** (A) Melanin content and (B) representative microscopic images of Mel-ab cells treated with 0.1 and 1 μM of altiratinib (ALT) or altiratinib analogs (ALT7a, ALT6a) in the absence or presence of FSK for 72 h. (Bar = 200 µm). (C) Microscopic images of the K14-SCF mouse-derived primary mouse melanocytes treated with altiratinib or altiratinib analogs (7a, 6a) (Bar = 200 µm).


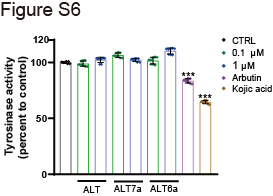


**Fig. S6.** **Short-term effect of altiratinib analogs on cellular tyrosinase activity.** Cellular tyrosinase activity of Mel-Ab cells after treatment with the vehicle (CTRL), altiratinib (ALT), or altiratinib analogs (ALT7a, ALT6a) for 2 h. Arbutin and kojic acid were used a positive control for the tyrosinase inhibitor.


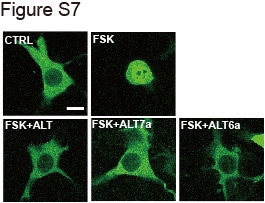


**Fig. S7** **Effects of altiratinib and altiratinib analogs on the subcellular localization of CRTC3 in B16F10 melanoma cells.** Representative microscopic images of the subcellular localization of CRTC3 1 h after treatment with altiratinib (ALT), altiratinib analogs (ALT7a, ALT6a), and forskolin (FSK) in B16F10 cells expressing CRTC3-EGFP. Bar = 10 μm


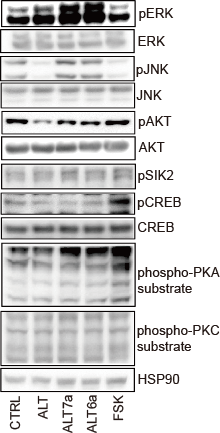


**Fig. S8** **Effects of altiratinib and altiratinib analogs on melanogenesis-relevant kinase activity in Mel-Ab cells.** The phosphorylation status of various kinases, as well as PKA and PKC activities, was examined after 1 hour of treatment with altiratinib (ALT), altiratinib analogs (ALT7a, ALT6a), and forskolin (FSK) in Mel-Ab cells.
